# Neurally plausible mechanisms for learning selective and invariant representations

**DOI:** 10.1186/s13408-020-00088-7

**Published:** 2020-08-18

**Authors:** Fabio Anselmi, Ankit Patel, Lorenzo Rosasco

**Affiliations:** 1 Center for Neuroscience and Artificial Intelligence Department of Neuroscience, Baylor College of Medicine, Baylor Plaza, 77030 Houston, USA; 2 Laboratory for Computational and Statistical Learning (LCSL), Istituto Italiano di Tecnologia, Genova, Via Dodecaneso, Genova, Italy; 3 Center for Brains, Minds, and Machines (CBMM), Massachusetts Institute of Technology, 43 Vassar Street, Cambridge, USA; 4 Department of Electrical & Computer Engineering, Rie University, 6100 Main St., 77005 Houston, USA

**Keywords:** Invariance, Hebbian learning, Group theory

## Abstract

Coding for visual stimuli in the ventral stream is known to be invariant to object identity preserving nuisance transformations. Indeed, much recent theoretical and experimental work suggests that the main challenge for the visual cortex is to build up such nuisance invariant representations. Recently, artificial convolutional networks have succeeded in both learning such invariant properties and, surprisingly, predicting cortical responses in macaque and mouse visual cortex with unprecedented accuracy. However, some of the key ingredients that enable such success—supervised learning and the backpropagation algorithm—are neurally implausible. This makes it difficult to relate advances in understanding convolutional networks to the brain. In contrast, many of the existing neurally plausible theories of invariant representations in the brain involve unsupervised learning, and have been strongly tied to specific plasticity rules. To close this gap, we study an instantiation of simple-complex cell model and show, for a broad class of unsupervised learning rules (including Hebbian learning), that we can learn object representations that are invariant to nuisance transformations belonging to a finite orthogonal group. These findings may have implications for developing neurally plausible theories and models of how the visual cortex or artificial neural networks build selectivity for discriminating objects and invariance to real-world nuisance transformations.

## Context and purpose of the study

How does the mammalian visual cortex build up object representations that are simultaneously selective for object identity and invariant to nuisance variation (e.g. changes in location, pose)? This is an old and challenging problem with a storied history of theoretical and practical attempts at solutions both in pattern recognition and computational neuroscience [1–12]. Much theoretical and experimental work [13–15] supports the hypothesis that most of the complexity of the object category recognition task is due to nuisance transformations such as pose, scale, and illumination. From this perspective, a natural property for a ventral stream representation to have is the ability to factor out task-nuisance variation (invariance) while still retaining task-relevant information (selectivity).

How to build such an architecture? Hubel and Wiesel’s seminal work [16–18] in studying cat visual cortex suggests an architectural solution that alternates between two cell types: simple cells, which detect features (selectivity); and complex cells, which integrate inputs from simple cells so as to provide robustness to small translations (invariance). This proposal provides a simple potential explanation for the structure and representational selectivity and invariance properties of the ventral stream, the part of the visual cortex that is believed to underlie the process of rapid object category recognition of images. Inspired by the work of Hubel and Wiesel, researchers in computer vision, theoretical and computational neuroscience, and machine learning have developed many architectures that embody this alternating motif: the neocognitron [2], HMAX [12], scale invariant feature transform (SIFT) [19], and most recently, deep convolutional neural networks (DCNNs). DCNNs are a class of architectures directly inspired by empirically observed properties of the visual cortex, and have proven to be very successful in learning representations for a wide variety of tasks that are simultaneously selective and invariant to nuisance transformations [20–27]. In particular, recent contributions on invariance and equivariance properties of DCNNs [28–32] are particularly relevant for our work although we focus on unsupervised Hebbian learning and emphasize neural plausibility.

The success of DCNNs in object recognition has revolutionized computer vision, audition and sparked a new movement in computational neuroscience as well. Surprisingly, recent work has shown that DCNNs trained solely for object recognition can predict cortical responses in macaque and mouse with unprecedented accuracy, especially in higher visual cortical areas [13]. Furthermore, these studies show that the DCNN’s high prediction accuracy depends critically on its ability to build invariance to *large* nuisance transformations, with competing models failing to be invariant, for example, to large out-of-depth rotations. This confirms the predictions from earlier theory and experimental work that invariance to nuisances is the main difficulty to be surmounted in object recognition.

Despite these successes, a rigorous theoretical understanding of these artificial and neuronal representations—how certain architectures can establish them and specifically how they produce the selectivity and nuisance invariance needed—remains poorly understood. There are several key issues that bedevil this effort. First, real-world nuisance transformations are high-dimensional, nonlinear, and can be quite complex. Currently, no simple mathematical characterization of them exists. Second, studying DCNN representations is complicated by the fact that DCNN learning is (a) discriminative, relying on access to large quantities of hand-labeled data, a luxury the brain lacks; and (b) relies heavily on algorithms like backpropagation which are neurally implausible.^1^ These issues make it difficult to relate any advances in understanding nuisance transformations or the selectivity/invariance of DCNN representations back to the brain.

Given the complexity of real-world nuisance transformations, it makes sense to start by studying smaller, simpler classes of analytically tractable nuisance transformations. In this vein, we focus on nuisance transformations that belong to a *group* (see Definition 1 in Sect. 2). Many real-world nuisance transformations belong to groups, including 2D/3D translations (changes in object location), 2D/3D rotations (changes in object or camera pose), scalings (changes in ambient lighting), and permutations (rearrangements of objects in a scene). However, it should be noted that groups do not exhaust all possible object-identity preserving nuisance transformations: for example, object deformations or a change in an object’s style or texture. Nevertheless, the major advantage of working with groups is that their mathematical structure is well understood, with many concepts and tools available for analysis. Our work thus uses the group structure, in particular that of finite orthogonal groups; later on we discuss potential ways to relax this constraint, which we leave to future work (see also the Appendix for some preliminary arguments).

In order to address the issues above, here we study a simple instantiation of an alternating architecture with an unsupervised learning rule, applied to a dataset of inputs that is generated by nuisance transformations belonging to the cyclic (abelian) or dihedral (non-abelian) group. We also consider a more realistic dataset composed of 2D rotations of natural image patches.

Our main contributions, extending the work in [25, 33] and [34], are to detail a neurally plausible mechanism for building a representation that is selective and invariant with respect to a class of nuisance transformations, namely those belonging to a finite orthogonal group. Our novel contributions can be summarized as follows:

- Theorem 1 shows how the group structure of the input is intimately related to the set of possible synaptic weights for the simple cells, under a broad class of unsupervised learning rules.
- Theorem 2 gives a simple mechanism by which a complex cell can aggregate simple cell inputs in order to produce representations that are invariant to a larger class of nuisance transformations beyond translations.
- Lemma 3.2 gives theoretical guarantees regarding the selectivity of a population of complex cells (i.e. their ability to discriminate different classes of images), under the assumption of a hard threshold nonlinearity.

## Theoretical background: groups, alternating architectures, and learning rules

### Input structure and transformations

As anticipated in the introduction, we are interested in understanding how neuronal properties relate to the structure of the visual input. In this vein, understanding the structure of the visual input is essential. We start by recalling the formal definition of a group.

#### Definition 1

A group $(\mathcal {G},\star)$ is a set of elements $\mathcal {G}$ with a binary composition rule ⋆ such that the following properties hold:

- Closure: composing two group elements results in another group element.
  $$\forall a,b\in \mathcal {G}, \quad\exists c\in \mathcal {G}\quad\textrm{s.t.}\quad a\star b=c. $$
- Identity: the identity element belongs to the group.
  $$\exists e\in \mathcal {G}\quad\textrm{such that} \quad\forall a\in \mathcal {G}, \quad e\star a=a\star e=a. $$
- Inverse: each group element has an inverse.
  $$\forall a\in \mathcal {G},\quad \exists a^{-1} \quad\textrm{such that}\quad a\star a^{-1}=e. $$
- Associativity:
  $$(a\star b)\star c = a\star(b\star c),\quad \forall a,b,c\in \mathcal {G}. $$

One of the simplest examples of a group is $\mathcal {R}_{N}$, the finite group of *N* rotations in the plane $\mathbb{R}^{2}$, whose elements are 2D rotation matrices of the form

$$R_{\theta_{i}} := \left [ \textstyle\begin{array}{c@{\quad}c} \cos(\theta_{i}) &\sin(\theta_{i}) \\ -\sin(\theta_{i}) & \cos(\theta_{i}) \end{array}\displaystyle \right ]\in \mathbb {R}^{2\times2},\quad \theta_{i}=i \frac{2\pi}{N}, i \in[N], $$

where $[N] := \{ 1,2,\ldots,N \}$. It is straightforward to verify that the set of matrices $\mathcal {R}_{N} := \{ R_{\theta_{i}} : i \in[N] \}$ together with the operation of $2 \times2$ matrix multiplication forms a group.

In this paper, we consider the input space to be the *d* dimensional vector space $X := \mathbb {R}^{d}$. We denote the transformation of a point $x\in X$ by the group element $g \in(\mathbb {R}^{d\times d}, \cdot)$ as the action of the matrix $g \in \mathcal {G}$ on the vector $x \in X$ i.e. $gx := g \cdot x$.

A key mathematical object in this context is that of an orbit. Let $\operatorname{Orb}_{\mathcal {G}}(x)$ denote the *orbit* of $x \in X$ with respect to the group $\mathcal {G}$, defined as the set of transformations of *x* over all elements of the group:

1 $$ \operatorname{Orb}_{\mathcal {G}}(x) := \{ gx : g \in \mathcal {G}\}. $$

For the group of plane rotations $\mathcal {R}_{N}$, the orbit of a vector $v \in \mathbb {R}^{2}$ is simply $\operatorname{Orb}_{\mathcal {R}_{N}}(v) := \{ R_{\theta_{i}}v : i \in[N] \}$, the set of all rotations of *v*. Orbits with respect to a group $\mathcal {G}$ (or $\mathcal {G}$-orbits) allow us to define an equivalence relation on the input space *X*, the essential ingredient for defining both invariance and selectivity.

#### Definition 2

(Input equivalence relation)

Two inputs $x,x'\in \mathbb {R}^{d}$ are equivalent with respect to a group $\mathcal {G}$ iff there exists a transformation in $\mathcal {G}$ that maps *x* to $x'$. Mathematically,

2 $$ x\sim x' \quad\Leftrightarrow\quad\exists g\in \mathcal {G}\quad \textrm{s.t.}\quad x=gx'. $$

In other words $x \sim x'$ iff *x*, $x'$ belong to the same $\mathcal {G}$-orbit i.e. $\operatorname{Orb}_{\mathcal {G}}(x) = \operatorname{Orb}_{\mathcal {G}}(x')$.

This equivalence relation induces a partition of the input space into disjoint orbits or equivalence classes i.e. $X = \bigcup_{c \in \mathcal {C}} X_{c}$ where $\mathcal {C}$ is the set of equivalence classes (or categories or orbits) induced by the nuisance group $\mathcal {G}$. Intuitively all inputs belonging to the same orbit of $\mathcal {G}$ will be considered the ‘same’ in the sense that they belong to the same category $c \in\mathcal {C}$. For example, in image classification, two images $x, x' \in X$ may both contain a dog and hence belong to the same category, namely $c = \textrm{DOG}$.

Another example is the group of 2D rotations: two images will be considered equivalent if there exists a 2D rotation that, when applied to one of the images, makes the two images equal.

In this work we further suppose that the group consists of unitary transformations i.e. $g^{-1}=g^{T}$, $\forall g\in \mathcal {G}$. In other words, we consider finite orthogonal groups.

Since the space of input is partitioned into different equivalence classes (orbits), we can now precisely define what it means for an input representation to be invariant and selective.

#### Definition 3

(Invariance and selectivity)

A function is invariant if it maps elements of the same equivalence class into the same object (e.g. a number or a vector) and it is selective if it maps elements of two different equivalence classes into two different objects.

The partition of the input space into equivalence classes/orbits as described above will be the main assumption in our work. More precisely:

#### Assumption

(Visual input structure)

Let $X=\mathbb {R}^{d}$ and let $\mathcal {G}$ be a finite orthogonal group. Suppose that the set of inputs *S* consists of a base set of *Q* distinct inputs $\{ x_{1},x_{2},\ldots,x_{Q}\}\in X$ and each of their $\mathcal {G}$-orbits $\operatorname{Orb}(x_{q}) = \{ g x_{q}: g \in \mathcal {G}\}$. Then we have

3 $$ S := \bigl\{ g_{i} x_{q} : i \in[N], q \in[Q] \bigr\} = \bigl\{ g x_{q} : g \in \mathcal {G}, q \in[Q] \bigr\} , $$

where $N := | \mathcal {G}|$ is the size of the group.

Throughout the rest of this paper we assume that the set of inputs is generated in this manner.

How plausible are these assumptions? A few remarks are in order before describing our network model. First, although finite orthogonal groups are a special subset of all image transformations, they constitute (a good approximation of) a large class of nuisance transformations with respect to which the representations in visual cortex are invariant. These include changes in position (object/camera translation), 2D size (object 3D size/proximity), and orientation (object/camera rotation). Second, although complete orbits are rarely available in real datasets, a large enough sample of orbits is sufficient for approximation in our model (see Sect. 3.3). Third, non-group transformations, which constitute by far the majority of real nuisance transformations, can be approximated by translations.^2^

We next describe our cortical model, inspired by the findings of Hubel and Wiesel, and the class of admissible learning dynamics for the simple cell synaptic weights.

### Alternating architecture of simple and complex cells

We adopt the simple-complex cells model of the visual system originally proposed by Hubel and Wiesel in the sixties [16]. The model consists of a hierarchical structure iterating the motif of simple and complex cells where a *simple cell* *s* computes the scalar product between the visual stimulus $x\in \mathbb {R}^{d}$ and the cell’s weights $w\in \mathbb {R}^{d}$ followed by a nonlinearity, $\sigma: \mathbb {R}\to \mathbb {R}$

4 $$ s(x)=\sigma \bigl[w^{T}x \bigr], $$

and a *complex cell* *c* linearly aggregates the responses of simple cells:

5 $$ c(x)=\sum_{i=1}^{N} \sigma \bigl[w_{i}^{T}x \bigr], $$

where we consider a set of *N* simple cells.

We suppose the early stage of the visual information processing to be done by units of simple-complex cells. In the next section we define the set of admissible learning dynamics for the simple cell synaptic weights *w*.

### Class of online unsupervised learning rules

We consider a broad class of online learning algorithms derived from an unsupervised loss function of the form

6 $$ \mathcal {L}\bigl(\{w_{i}\}_{i=1}^{N}, \{x_{j}\}_{j=1}^{R=Q|\mathcal {G}|} \bigr)=\mathcal {L}(W,S)= \sum _{i,j} f \bigl(\sigma \bigl[w^{T}_{i}x_{j} \bigr] \bigr), $$

where $w_{i}=W_{(:,i)}$ are the synaptic weights of the simple cells, $x_{j}\in S$ are the inputs, and $f:\mathbb {R}\to \mathbb {R}_{+}$ is a Lipschitz function. In general *N* is a free parameter, but here and in simulations we fix *N* to be the orbit size i.e. one possible degenerate solution. Unsupervised loss function of the form above includes i.e. Hebbian, Oja’s, Ica, and Foldiack [35].

In particular, for the simulations we used the loss $f(\cdot)=(\cdot )^{2}$ and a Heaviside nonlinearity $\sigma= H(\cdot-z)$ with a big fixed negative threshold *z* to use the full range of activations (see also the Appendix and Sect. 3.2):

7 $$\begin{aligned} \begin{aligned}[b]\mathcal {L}\bigl(\{w_{i}\}_{i=1}^{N}, \{x_{j}\}_{j=1}^{R=Q|\mathcal {G}|} \bigr) &= \sum _{i,j} \bigl(\sigma \bigl(w^{T}_{i}x_{j} \bigr) \bigr)^{2} \\ &=\operatorname{Tr} \bigl(\sigma \bigl(W^{T}S \bigr)\sigma \bigl(S^{T}W \bigr) \bigr) \\ &=\bigl\Vert \sigma \bigl(W^{T}S \bigr)\bigr\Vert _{F}^{2}.\end{aligned} \end{aligned}$$

The online update rule for the *i*th simple cell’s weights is obtained deriving Eq. (6) w.r.t. $w_{i}$:

8 $$ \Delta_{t} w_{i} = w^{t+1}_{i}-w^{t}_{i}\propto \nabla_{w_{i}}\mathcal {L}(W,S)= \sum_{j} f' \bigl(\sigma \bigl[w^{t,T}_{i}x_{j} \bigr] \bigr)\sigma' \bigl[w^{t,T}_{i}x_{j} \bigr]x_{j}, $$

where, in simulations, the initial weights were initialized at random. In words: any learning associated with a smooth loss of the simple cells response is admissible.

After $t^{*}$ updates the simple cell weights will be

9 $$ w^{t^{*}}_{i}=-\alpha\sum _{t=1}^{t^{*}} \sum_{j} f' \bigl(\sigma \bigl[w_{i}^{t,T}x_{j} \bigr] \bigr)\sigma' \bigl[w_{i}^{t,T}x_{j} \bigr]x_{j}, $$

where the initial cell’s weights $w_{0}$ are chosen to be the zero matrix for simplicity and $\alpha\in \mathbb {R}_{+}$ is the learning rate.

This assumption, together with that of the input structure (Eq. (3)), will be enough to derive a characterization of the learned weights for the simple cells (Theorem 1).

### Learning simple cells and how to aggregate simple cells

Before going into the mathematical details of the next section, we first give an intuition and describe a possible biological mechanism for the learning of simple cells’ receptive fields and simple cells’ aggregation operated by a complex cell. The idea is to consider two phases of neuronal plasticity.

In the first phase, Hebbian learning will tune a simple cell’s receptive field to any of the possible degenerate solutions of the weights dynamical system. The degeneracy is due to the transformations in the set of inputs (e.g. natural images rotations).

In the second phase, a Hebbian-type hypothesis on the behavior of a complex cell will be employed: cells that maximally fire in the presence of input belonging to the same class/category will be wired together by a complex cell. The idea is that the weights of a population of simple cells maximally firing over a collection $\mathcal {G}$-orbits form an orbit. In the next section, we formalize this idea and provide proofs of key results.

## Theoretical results: selectivity and invariance of image representations

In the following we present the mathematical proofs for the learning and aggregation mechanism explained in the previous section. We proceed by steps:

1. First, we show that the structure of the visual input implies that if *w* is a possible solution for the simple cell’s weights dynamics so is each element of its equivalence class, the orbit $\operatorname {Orb}_{\mathcal {G}}(w)$.
2. Second, we prove how a Hebbian type of learning can account for a biologically plausible mechanism to aggregate simple cells to obtain a complex cell invariant representation.
3. Finally, we prove how simple cells with “enough” random thresholds nonlinearities provide a way to implement a selective representation.

Summarizing, our main contribution is as follows.

### Result

(Main, informal)

Suppose that the set of inputs is a collection of group transformations of images as in Eq. (3). Suppose the simple-complex cells model of Hubel and Wiesel and a learning dynamics as in Eq. (9). Then the complex cell response is invariant and selective with respect to the group transformations.

### Learning invariance

As explained above, the presence of equivalence classes (symmetries) in the stimulus space produces many equivalent possibilities for the simple cells learned weights (degeneracy of the solution space). In particular, an orbit of a solution is itself a set of solutions. More precisely:

#### Theorem 1

(Possible simple cells learned weights)

*Let the set of inputs be composed by a collection of group transformations of images as in Eq*. (3). *Let the learning rule be admissible in the sense of Eq*. (9). *Then if* $w^{*}$ *is a possible solution for the learned simple cell weights at time* $t^{*}=k|\mathcal {G}|$, $k\in\mathbb{N}$, *so is* $gw^{*}$ *for all* $g\in \mathcal {G}$.

#### Proof

We want to prove that if $w^{*}$ is a solution, then $gw^{*}$ is. Under a transformation $g\in \mathcal {G}$ of the weight $w_{i}$, $w_{i}\to gw_{i}$, each of the addends in Eq. (9) transforms as follows:

$$\begin{aligned} \Delta w_{i} =&\nabla_{w_{i}} \mathcal {L}\bigl(\{ w_{1},\ldots,gw_{i},\ldots,w_{N}\} ,S \bigr) \\ =& \sum_{j} f' \bigl(\sigma \bigl[(gw_{i})^{T}x_{j} \bigr] \bigr)\sigma ' \bigl[(gw_{i})^{T}x_{j} \bigr]x_{j} \\ =& g\sum_{j} f' \bigl(\sigma \bigl[w_{i}^{T}g^{T}x_{j} \bigr] \bigr)\sigma' \bigl[w_{i}^{T}g^{T}x_{j} \bigr]g^{T}x_{j} \\ =& g\nabla_{w_{i}}\mathcal {L}(W,S), \end{aligned}$$

where in the second line we inserted the identity $e=gg^{T}$. The last line follows noting that: (1) by the closure property of the group, $g^{T}x_{j}$ is an element of the orbit of $x_{j}$; (2) since $t^{*}=k|\mathcal {G}|$, all the first *k* orbit elements of the input *S* are present in the sum, the sum is invariant. The last equation implies that if $w^{*}$ is a solution, so is $gw^{*}$ for all elements of the group. □

The theorem proves that orbits of weights are possible solutions among all solutions of the learning dynamics.

Suppose now that the set of simple cells are mature after the first phase of synaptic plasticity and their weights are fixed. A natural question is then: which set of simple cells is a complex cell going to aggregate? As informally mentioned in the previous section, if we assume that a complex cell aggregates simple cells that fire together, then we can deduce that the aggregated cells have weights that form an orbit. More precisely:

#### Theorem 2

(Complex cells pooling and invariance)

*A complex cell learns to aggregate over simple cells whose weights form an orbit with respect to the group* $\mathcal {G}$. *Furthermore*, *its response is invariant with respect to* (*nuisance*) *transformations from the group* $\mathcal {G}$.

#### Proof

Let $E_{\textrm{simple}}$ be the set of all possible weights for simple cells after learning. This set is determined by the learning dynamics given by Eq. (9). Let the new incoming stimulus set $S_{\mathrm{new}}=\{ g_{1}s_{1},\ldots,g_{|\mathcal {G}|}s_{1},\ldots,g_{1}s_{M},\ldots,g_{|\mathcal {G}|}s_{M}\}$ be composed of transformations of a new input set in agreement with our input assumption in Eq. (3).

Let *w̄* be the weights of the simple cell that maximally respond to the $S_{\mathrm{new}}$ input i.e.

10 $$ \bar{w} = \mathop {\operatorname {arg\,max}}_{w\in E_{\textrm{simple}}} \sum _{j}f \bigl(\sigma \bigl(w^{T}s^{\mathrm{new}}_{j} \bigr) \bigr). $$

Which other simple cells a complex cell will aggregate to the simple cells with *w̄* weights? The key observation is that the sum in Eq. (10) is invariant to a transformation $\bar {w}\rightarrow g\bar{w}$, $\forall g\in \mathcal {G}$. Thus we have

11 $$ w_{2} = \mathop {\operatorname {arg\,max}}_{w\in E_{\textrm{simple}},w\neq\bar{w}} \sum_{j}f \bigl(\sigma \bigl(w^{T}s^{\mathrm{new}}_{j} \bigr) \bigr)=g \bar{w} $$

for some $g\in \mathcal {G}$. The reasoning can be repeated leading to $w_{i}=g_{i}\bar{w}$, $w_{1}=\bar {w}$. Note that elements of the same orbit can be repeatedly sampled in this way. However, as this does not impact our results, we assume for simplicity that the selected weights form an orbit and not multiple copies of it. The invariance property of the complex cell response follows from the group property of closure:

12 $$\begin{aligned} c(gx) =&\sum_{i=1}^{|\mathcal {G}|} \sigma \bigl[(gx)^{T}g_{i}\bar{w} \bigr]=\sum _{i=1}^{|\mathcal {G}|}\sigma \bigl[ x^{T}g^{T}g_{i} \bar{w} \bigr] \\ =&\sum_{i=1}^{|\mathcal {G}|}\sigma \bigl[x^{T}\hat{g}_{i}\bar{w} \bigr]=c(x), \end{aligned}$$

where we relabeled the group elements as $\hat{g}_{i}=g^{T}g_{i}$. □

This result gives an explanation for how a simple-complex model of visual information processing, together with Hebbian-type learning, can provide an input representation that is invariant to a larger class of nuisance transformations, beyond translations.

### Selectivity

Although invariance is necessary, it is not sufficient: indeed we can think of trivially invariant representations e.g. a function that maps all inputs to 0. Selectivity, the ability to separate/discriminate different equivalence classes of inputs, is the other important property.

In the following we show the importance of the presence of a nonlinear function in the simple cell response for the selectivity property. In particular we analyze the case of simple cells with nonlinearity given by the Heaviside function with threshold $z\in \mathbb {R}$ i.e. we consider a family of nonlinearities $\{\sigma_{z}(\cdot)\equiv H(\cdot-z), z\in \mathbb {R}\} $. The complex cell response is in this way modeled as a family of responses $\{c_{z}\}_{z\in \mathbb {R}}$ indexed by the variable *z*:

13 $$ c_{z}(x)\equiv \bigl(c(x) \bigr)_{z}= \sum_{i=1}^{|\mathcal {G}|}H \bigl(x^{T}g_{i}w-z \bigr),\quad z\in \mathbb {R}. $$

Next we prove that allowing for the thresholds *z* to be in a continuous range produces a selective complex cell response. More precisely:

#### Lemma

(Complex cells selectivity)

*Let* $x,x'\in \mathbb {R}^{d}$ *be two inputs and* $c(x)$, $c(x')$ *be the complex cell response as in Eq*. (13). *Then the distance defined as*

14 $$ \operatorname{dist} \bigl(x,x' \bigr):=\bigl\Vert c(x)-c \bigl(x' \bigr)\bigr\Vert _{\ell_{2}} $$

*is zero iff* $x\sim x'$.

#### Proof

Let $A := \{x^{T}g_{i}w, i=1,\dots,|\mathcal {G}|\}$ and $B := \{(x')^{T}g_{i}w, i=1,\dots,|\mathcal {G}|\}$ be the sets containing the simple cells’ responses to inputs *x*, $x'$, respectively. Note first that the effect of a transformation of the input $x \to gx$ on the sets *A*, *B* is a permutation of their elements. This is due to the closure property of the group $\mathcal {G}$. Second, note that $c_{z}(x)$ is the value, at *z*, of the cumulative distribution function (CDF) of the simple cells’ responses to the stimulus *x*. To conclude the proof, we recall that the CDF is a maximal invariant with respect to the permutation group [36]. This means that the distance between the CDFs of *A*, *B* is zero iff the simple cells’ responses for *x* and $x'$ differ by a permutation. In other words $x\sim x'$ iff $\operatorname{dist}(x,x')=0$. □

**Figure 1:**
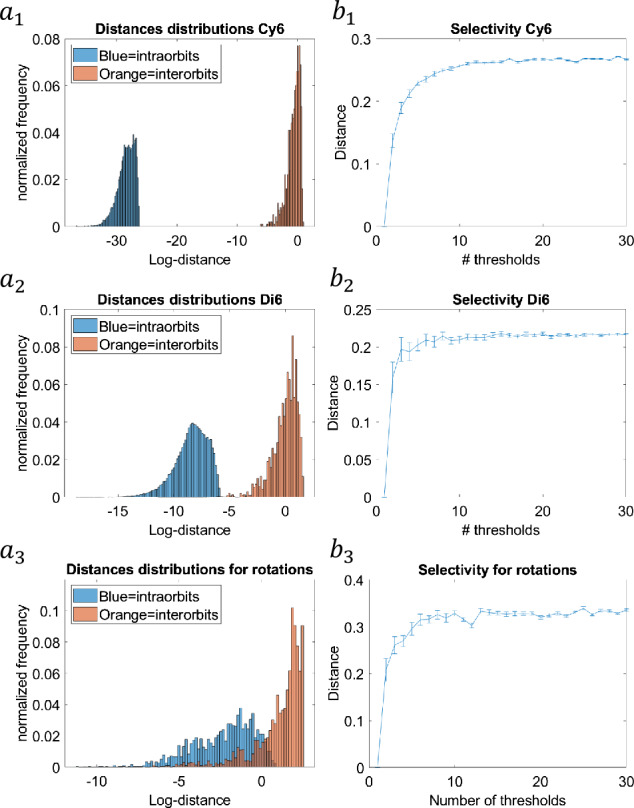
Invariance and selectivity: ($\boldsymbol{a_{1}}$, $\boldsymbol{a_{2}}$, $\boldsymbol{a_{3}}$) intra- and inter-orbits log distances statistic for toy data (cyclic ($a_{1}$) and dihedral group ($a_{2}$)) and rotated natural patches ($a_{3}$); ($\boldsymbol{b_{1}}$, $\boldsymbol{b_{2}}$, $\boldsymbol{b_{3}}$) selectivity in terms of cosine similarity for the three datasets with respect to the number of Heaviside nonlinearities

Intuitively the selectivity property, which is partially lost by the complex aggregation operation, can be recovered by allowing different nonlinearities in simple cells. The continuous set of thresholds is clearly an implausible biological assumption. However, a weaker result can be obtained by sampling (uniformly at random) the set of thresholds and applying a concentration inequality (see Sect. 3.3). Experimental evidence is given in Fig. 1($a_{2}$, $b_{2}$, $c_{2}$).

One possible biological interpretation/implementation for our model could be the following. Let us consider a complex cell corresponding to a pyramidal cell. Let us assume that there are subunits on the dendritic tree of the cell, each one receiving similar inputs. Let us also assume that the subunits are electrically separate in the sense that each of them has the ability to produce dendritic spikes. Then effectively each subunit will be equivalent to a simple cell tuned to different ($w_{i}$) weights: this is because of the degeneracy of the solutions to the dynamical system, as described in Sect. 3. Moreover, each simple cell will have very similar thresholds: this is because receiving the same input the range of their responses will be approximately equal. The soma of the pyramidal cell will summate the simple-cell-like subunit activities. Literature supports the hypothesis of computational subunits e.g. [37].

### Approximate invariance and selectivity

In a real scenario we could not count on an infinite number of thresholds and input data will not consist of full orbits as in Assumption 2.1. However, we show here that the results in Sect. 3 still hold in expectation with high probability.

More precisely the result in Lemma 3.2 can be obtained in expectation, for a finite number of thresholds, using a simple concentration inequality e.g. Hoeffding’s inequality. Let

$$\hat{c}(x)=\sum_{q=1}^{Q}c_{z_{q}}(x), $$

where $z_{q}$ is sampled uniformly at random in the range of the simple cell responses. Applying Hoeffding’s inequality, we have

$$ \Pr \bigl\{ \bigl\vert \operatorname{dist} \bigl(x,x' \bigr)- \hat{ \operatorname{dist}} \bigl(x,x' \bigr) \bigr\vert >\epsilon \bigr\} < 2e^{-\frac{Q\epsilon^{2}}{2p}}\quad \forall\epsilon> 0, $$

where $\hat{\operatorname{dist}}(x,x')={\hat{c}(x)-\hat{c}(x')}$. By choosing the number of complex cells *Q* to be sufficiently large, we obtain, in expectation, a very good approximation of the true distance.

Clearly, because of the restricted dynamic range of cortical cells, the number thresholds is likely to be small. However, related representations are possible using other classes of nonlinearities. Although a CDF is fully represented by *all* of its moments, in practice often just a few moments—such as the average, energy (second moment), or max (∞ moment)—can serve as an effective replacement. Also note that any linear combination of the moments is also invariant, and so a small number of linear combinations is likely to be sufficiently selective.

A similar argument can be made for approximating the loss in Eq. (6) or the update rule in Eq. (8) when the complete set of input orbits is not available (violating the key Assumption 2.1).

## Experimental results

We tested the proposed model for invariance and selectivity of the complex cell output on an artificial input set and a natural image dataset.

For the artificial dataset the group of transformations $\mathcal {G}$ was chosen to be a permutation group acting on a vector space of dimension 6. We considered the cyclic group (abelian) or the dihedral group (non-abelian). The input sets (*S*, $S_{\mathrm{new}}$) were generated by picking random vectors (uniformly sampled from the unit ball in $\mathbb {R}^{6}$) and transforming them according to all transformations of the selected permutation group.

To have a more realistic dataset, we then considered natural images. We extracted same size patches (of radius 10 pixels) at random on the natural image and rotated each patch according to a finite group of rotations (six equally spaced rotation angles).

Figure 1 summarizes our findings. To test the properties of invariance and selectivity for the artificial input datasets, we produced two sets of orbits: the input set *S* (100 orbits) and the new input $S_{\mathrm{new}}$ (2 orbits). This applies for both the cyclic and the dihedral group.

Similarly, for the natural images dataset, we produced a dataset *S* of 100 random extracted patches and their rotations and the new input $S_{\mathrm{new}}$ (two orbits).

For both datasets, we learned the simple cells weights $U^{*}$ and the complex cell aggregation operation by maximizing, respectively, their response to *S* and $S_{\mathrm{new}}$ running the maximization problem:

15 $$ U^{*}=\mathop {\operatorname {arg\,max}}_{U\in \mathbb {R}^{d\times N}} \bigl\Vert \sigma \bigl(U^{T}S \bigr)\bigr\Vert ^{2}_{F}+\lambda \bigl\Vert \sigma \bigl(U^{T}S_{\mathrm{new}} \bigr)\bigr\Vert ^{2}_{F} \quad\text{s.t.}\quad U^{T}U=\mathrm{Id} $$

with $\lambda\in \mathbb {R}_{+}$ (see also the Appendix).

We then calculated the coding operated by the complex cell as in Eq. (13) for a few numbers of random thresholds (10) and calculated the code distances for couples of inputs belonging to the same orbit (equivalent class, intra-orbit) or different orbits (not equivalent, inter-orbits). In more detail, we calculated the distance $\operatorname {dist}:R^{d\times d}\to R_{+}$:

$$ \operatorname{dist}(x,y)=\sum_{z} \operatorname{dist}_{z}(x,y)= \sum_{z} \bigl\vert c_{z}(x)-c_{z}(y) \bigr\vert =\sum _{z} \Biggl\vert \sum _{i=1}^{ \vert \mathcal {G}\vert }\sigma_{z} \bigl(x^{T}u^{*}_{i} \bigr)-\sum _{i=1}^{ \vert \mathcal {G}\vert }\sigma_{z} \bigl(y^{T}u^{*}_{i} \bigr) \Biggr\vert . $$

In the case of elements belonging to the same orbit i.e. $x,y\in \operatorname{Orb}(x)=\operatorname{Orb}(y)$, we expect the distance to be zero (or approximately zero in simulations) due to the invariance properties. Otherwise different from zero.

Figure 1($a_{1}$, $b_{1}$, $c_{1}$) represents the distribution of distances $\operatorname{dist}(x,y)$ when $x\sim y$ i.e. they belong to the same orbit (blue histogram) or when $x\nsim y$ i.e. they do not belong to the same orbit (orange histogram).

As expected, the distribution of distances among elements within the same equivalence class (same orbit) and those among different classes of equivalence (different orbits) are significantly different. Statistics was done for 1000 orbits test-set for the two artificial datasets and the natural images dataset. Log plot of distances is shown for the reader’s convenience.

Finally, Fig. 1 ($a_{2}$, $b_{2}$, $c_{2}$) shows how the separation among different classes of equivalent images behaves with respect to the simple cell nonlinearity. We analyzed the case of a Heaviside-threshold nonlinearity plotting the cosine inter-distance among two random orbits against the number of (random) thresholds used in computing the complex cells response for the three datasets. The plot shows how the cosine similarity grows with the number of thresholds eventually reaching a plateau.

Taken together, the experimental results confirm our theoretical results both for toy model and natural images patches, although in the second case the overlap between the distribution of intra- and inter-distances is much more marked.

## Conclusions, implications, and future work

In this report, extending the work in [25, 33], and [34], we used tools from group theory and invariant theory, together with insights from the neuroscience of the visual cortex, to develop a forward model of visual recognition.

Under weak assumptions on the neurons learning dynamics, we showed how the simple Hubel–Wiesel model of early visual cortex can automatically account for nontrivial invariance and selective properties of the visual information processing.

Our contribution is relevant for any data in high-dimensional perceptual spaces that have a low-dimensional intrinsic structure (e.g. transforming objects or sounds). The preliminary work outlined here focused for simplicity on low-dimensional permutation groups and rotation groups, but it defines a mathematical framework that opens to natural extensions. One intriguing direction is that of non-group transformations which constitute by far the majority of real object transformations. The idea is that, if we assume that the object transformations define a smooth manifold, locally, a Lie group is defined by the generators on the tangent space (one important example is rotations in depth, where 3*D* rotations are projected into a 2*D* space by the retina). This allows the complex global transformation to be decomposed into a collection of local object transformations that obey a group structure. This is also the concept of hierarchical-compositional networks (like DCNNs) where complex global transformations are decomposed into a hierarchy of simple, local ones. Finally, our model predictions strongly depend on the visual input structure i.e. group transformations of objects: however, if on one side this might be seen as a weakness, on the other it is a great opportunity to design ad hoc artificial input to test the model predictions in real neurophysiological experiments.

## Appendix

### Approximate invariance for non-group transformations (from [38])

In here we briefly discuss extensions of this work for getting an approximately invariant signature for transformations that do not have a group structure. In fact, most realistic signal transformations will not have a group structure. However, assuming that the transformation defines a smooth manifold, we have (by the theory of Lie manifolds) that locally a Lie group is defined by the generators on the tangent space. We illustrate this in a simple example.

Let $x\in X \subseteq \mathbb {R}^{d}$ and $s:\mathbb {R}^{d} \times \mathbb {R}^{P} \rightarrow \mathbb {R}^{d}$ be a $C^{\infty}$ transformation depending on $\varTheta=(\theta_{1},\dots,\theta_{P})$ parameters. For any fixed $x\in X$, the set $M =(s(x,\varTheta), \varTheta\in \mathbb {R}^{P})$ describes a differentiable manifold. If we expand the transformation around e.g. $\vec{0}$, we have

16 $$ s(x,\varTheta) = s(x,\vec{0}) + \sum _{i=1}^{P}\frac{\partial s(x,\varTheta)}{\partial\theta_{i}} \theta_{i} + o \bigl(\bigl\Vert \varTheta\bigr\Vert ^{2} \bigr) = x + \sum _{i=1}^{P}\theta_{i} L_{\theta_{i}}(x) + o \bigl(\bigl\Vert \varTheta\bigr\Vert ^{2} \bigr), $$

where $L_{\theta_{i}}$ are the infinitesimal generators of the transformation in the *i*th direction.

Therefore locally (when the term $o(\Vert\varTheta\Vert^{2})$ can be neglected) the associated group transformation can be expressed by exponentiation as follows:

$$g(\varTheta) = \exp(\theta_{1}L_{\theta_{1}}+ \theta_{2}L_{\theta_{2}}+\cdots+\theta_{P}L_{\theta_{P}}). $$

In other words, instead of a global group structure of the transformation we will have a collection of local transformations that obey a group structure. Thus in this light the local learned weights will be orbits w.r.t. the local group approximating the non-group global transformation.

### Simple cells weights complex cells pooling learning

To mimic in the computational experiments the behavior of simple and complex cells as described by Theorem 2, we formulated the learning problem as follows: find the matrix $U^{*}$ (whose columns are the learned simple cells weights) such that

17 $$ U^{*}=\mathop {\operatorname {arg\,max}}_{U\in \mathbb {R}^{d\times d}} \bigl\Vert \sigma \bigl(U^{T}S \bigr)\bigr\Vert ^{2}_{F}+\lambda\bigl\Vert \sigma \bigl(U^{T}S_{\mathrm{new}} \bigr)\bigr\Vert ^{2}_{F}\quad \text{s.t.}\quad U^{T}U=\mathrm{Id}, $$

where *S* is the set of inputs presented to the simple cells (as in Eq. (3)) to learn their weights and $S_{\mathrm{new}}$ is the new input set, a new set of orbits.

We employed a Heaviside nonlinearity with fixed negative threshold to use the full range of neural activations.

To find a solution, we applied a gradient descent approach. We found the best results are with $\lambda=10^{-2}$ (artificial dataset) and $\lambda=10^{-1}$ (natural images) with a constant learning of 10^−7^. Note that differently from what was described as a two-phase learning in the main text, we solved a joint optimization problem for *S*, $S_{\mathrm{new}}$. Although biologically plausible, the two-phase learning is not efficient since phase one consists of learning a very overcomplete dictionary *U*, a known hard optimization problem.

## Footnotes

1 Recent works (e.g. [39]) have attempted to develop forms of backpropagation that avoid weight transport, the most implausible aspect of the algorithm. However, to date there is no empirical evidence to suggest that the brain uses these algorithms.

2 This can be seen via a Taylor expansion, assuming the transformations satisfy certain local regularity/smoothness conditions; see the Appendix for a more detailed argument.

## Abbreviations

DCNN: deep convolutional neural network
SIFT: scale invariant feature transform

## References

[1] McCulloch WS, Pitts W (1943). A logical calculus of the ideas immanent in nervous activity. Bull Math Biophys.

[2] Fukushima K (1980). Neocognitron: a self-organizing neural network model for a mechanism of pattern recognition unaffected by shift in position. Biol Cybern.

[3] Caelli TM, Liu Z-Q (1988). On the minimum number of templates required for shift, rotation and size invariant pattern recognition. Pattern Recognit.

[4] Lenz R (1990). Group invariant pattern recognition. Pattern Recognit.

[5] Földiák P (1991). Learning invariance from transformation sequences. Neural Comput.

[6] Grace AE, Spann M (1991). A comparison between Fourier–Mellin descriptors and moment based features for invariant object recognition using neural networks. Pattern Recognit Lett.

[7] Flusser J, Suk T (1993). Pattern recognition by affine moment invariants. Pattern Recognit.

[8] Olshausen BA, Anderson CH, Van Essen DC (1995). A multiscale dynamic routing circuit for forming size- and position-invariant object representations. J Comput Neurosci.

[9] Van Gool L, Moons T, Pauwels E, Oosterlinck A (1995). Vision and Lie’s approach to invariance. Image Vis Comput.

[10] Michaelis M, Sommer G (1995). A Lie group approach to steerable filters. Pattern Recognit Lett.

[11] Wood J (1996). Invariant pattern recognition: a review. Pattern Recognit.

[12] Riesenhuber M, Poggio T (1999). Hierarchical models of object recognition in cortex. Nat Neurosci.

[13] Yamins DLK, Hong H, Cadieu CF, Solomon EA, Seibert D, DiCarlo JJ (2014). Performance-optimized hierarchical models predict neural responses in higher visual cortex. Proc Natl Acad Sci USA.

[14] Pinto N, Cox DD, DiCarlo JJ (2008). Why is real-world visual object recognition hard?. PLoS Comput Biol.

[15] Lee T, Soatto S (2012). Video-based descriptors for object recognition. Image Vis Comput.

[16] Hubel DH, Wiesel TN (1962). Receptive fields, binocular interaction and functional architecture in the cat’s visual cortex. J Physiol.

[17] Hubel DH, Wiesel TN (1965). Receptive fields and functional architecture in two nonstriate visual areas (18 and 19) of the cat. J Neurophysiol.

[18] Hubel DH, Wiesel TN (1968). Receptive fields and functional architecture of monkey striate cortex. J Physiol.

[19] Lowe DG (1999). Object recognition from local scale-invariant features. Proceedings of the seventh IEEE international conference on computer vision.

[20] Achille A, Soatto S (2017). Emergence of invariance and disentangling in deep representations. ICML workshop on principled approaches to deep learning.

[21] Soatto S. Steps towards a theory of visual information: active perception, signal-to-symbol conversion and the interplay between sensing and control. arXiv:1110.2053 (2011).

[22] Lessmann M, Würtz RP (2014). Learning invariant object recognition from temporal correlation in a hierarchical network. Neural Netw.

[23] Lenc K, Vedaldi A (2015). Understanding image representations by measuring their equivariance and equivalence. IEEE conf. on computer vision and pattern recognition (CVPR).

[24] Shao Z, Li Y (2015). Integral invariants for space motion trajectory matching and recognition. Pattern Recognit.

[25] Anselmi F, Rosasco L, Poggio T (2015). On invariance and selectivity in representation learning. Inf Inference.

[26] Cohen TS, Welling M (2016). Group equivariant convolutional networks. International conference on machine learning (ICML).

[27] Gens R, Domingos PM (2014). Deep symmetry networks. Advances in neural information processing system (NIPS).

[28] Anderson BM, Hy T, Kondor R (2019). Cormorant: covariant molecular neural networks. Advances in neural information processing systems.

[29] Cohen TS, Geiger M, Weiler M (2019). A general theory of equivariant CNNs on homogeneous spaces. Advances in neural information processing systems.

[30] Kondor R, Trivedi S (2018). On the generalization of equivariance and convolution in neural networks to the action of compact groups. Proceedings of the 35th international conference on machine learning, ICML 2018.

[31] Kondor R, Lin Z, Trivedi S. Clebsch–Gordan nets: a fully fourier space spherical convolutional neural network. arXiv:1806.09231 (2018).

[32] Cohen TS, Geiger M, Köhler J, Welling M. Spherical CNNs. arXiv:1801.10130 (2018).

[33] Anselmi F, Leibo JZ, Rosasco L, Mutch J, Tacchetti A, Poggio T (2016). Unsupervised learning of invariant representations. Theor Comput Sci.

[34] Anselmi F, Evangelopoulos G, Rosasco L, Poggio T (2019). Symmetry-adapted representation learning. Pattern Recognit.

[35] Hassoun MH (1995). Fundamentals of artificial neural networks.

[36] Zacks S (1971). The theory of statistical inference.

[37] Polsky A, Mel BW, Schiller J (2004). Computational subunits in thin dendrites of pyramidal cells. Nat Neurosci.

[38] Anselmi F, Evangelopoulos G, Rosasco L, Poggio T. Symmetry regularization. CBMM Memo 063 (2017).

[39] Akrout M, Wilson C, Humphreys PC, Lillicrap TP, Tweed DB. Deep learning without weight transport. arXiv:1904.05391 (2019).

